# Polymeric Biomass Derived Adsorbents for Co(II) Remediation, Recycling and Analysis

**DOI:** 10.3390/polym14091647

**Published:** 2022-04-19

**Authors:** Lavinia Tofan

**Affiliations:** Department of Environmental Engineering and Management, “Cristofor Simionescu” Faculty of Chemical Engineering and Environmental Protection, “Gheorghe Asachi” Technical University of Iasi, 73 D. Mangeron Blvd, 700050 Iasi, Romania; lavinia_tofan@yahoo.com

**Keywords:** polymeric biomass, biosorption, cobalt, removal, recovery, analysis, real samples

## Abstract

The gradual replacement of conventional materials with materials tailored to the green development goals is one of the needs of the day. Correspondingly, this article reviews and integrates, for the first time, the gathered knowledge on the use of the adsorbents based on polymeric biomasses (biosorbents) for a cleaner separation of cobalt (Co) from synthetic and actual solutions. It is a two-part comprehensive approach that debates the Co biosorption potential of bio-based polymers from the perspective of their virtual and real applications for decontamination, recovery, and analytical purposes. First, the removal performances of these materials to batch and fixed column biosorption of Co(II) from mono-component and multi-metallic laboratory solutions are systematized and discussed. Following that, the focus of the first part is shifted to the analytical capabilities of the biosorbents proposed for Co(II) quantification from synthetic solutions. The second section considers the polymeric biomasses successfully incorporated in practical strategies for the removal and recovery of Co(II) from real solutions. The opportunities provided by the use of biosorbents for the development of accurate and greener procedures in Co(II) analysis are also highlighted. The directions in which the research on this topic should be continued and strengthened are suggested.

## 1. Introduction

The element of interest for this work, namely cobalt (Co), which may exist in the 0, +2, and +3 states of oxidation, has many common features with other members of the heavy metals family to which it belongs, but also radioactive properties [1]. It is ranked as a critical metal [2] and, depending on its concentration level, can act both as a priority pollutant and an essential element for metabolic activities [3]. Taking into account the prevalence of this form in environmental conditions, divalent cobalt, Co(II) receives the most attention.

Co falls currently into the category of critical materials on the basis of its economic significance and the risk of supply shortcomings [4]. Besides the notorious uses in rechargeable lithium-ion batteries and super alloys, Co is also critical for plenty of industries, such as hydrometallurgical, electroplating, petrochemical, electronics, and ceramics, as well as for nuclear power plants, and medicine [5,6,7]. This intensive Co utilization can cause natural resources depletion. On the other hand, the wide spectrum of Co applications results in a continuous aggravation of its pollution impact and more and more serious problems in public health [8]. Therefore, the remediation of Co contaminated aqueous media is an important contemporary society task. Having as main objective the meeting of ever-increasing demand for Co, which is estimated at 183% in 2030 [9], the recovery of Co from waste solutions is also beneficial for environmental protection. One other benefit is the contribution of recovered Co use to the reduction of CO_2_ generation [10]. All these aspects highlight the key role of an efficient method of separation/preconcentration that is able to cope with the requirements imposed for complete removal, quantitative recovery, and accurate analysis of Co from different effluents and sources.

The approaches developed for the above mentioned goals focus on separation methods of Co from aqueous solutions, such as adsorption [11,12,13], ion exchange [14,15], chemical precipitation [16,17], membrane processes [18], solvent extraction [19,20] and solid-phase extraction [21]. However, the applicability of adsorption predominates over all the other conventional methods [22,23]. The adsorption popularity is due, to some extent, to the rise of its sustainable variant, known as biosorption, and is considered an innovative tool of the 21st century technology of separation [24]. The driving force of the booming interest in the biosorption process is represented by the easily available, renewable, and recyclable polymer materials engaged as biosorbents [25,26,27,28,29,30], and is characterized by unicity in diversity. An impressive number of critical reviews emphasized the high capability of biological materials to develop biosorption-based approaches for removal-recovery of heavy metals from liquid effluents [31,32,33,34,35,36,37,38,39,40] and their analysis from a wide range of samples [41,42,43,44,45,46]. At the same time, their common recommendation is that, in order to promote the transition from virtual applications to practical applications, the strength of the biosorption potential must be confirmed in the context of real situations.

Despite the fact that Co(II) is regarded as a good model pollutant for research [47], it has been very rarely addressed in the review articles on biosorption topic and is mostly oriented towards other heavy metals, such as Cu, Zn, Cd, and Pb. The opportunities and constraints of the use of 11 categories of adsorbents, including those based on natural materials, agricultural waste materials, and biopolymers for Co(II) uptake from contaminated waters, were pointed out [22]. The attributes of filamentous fungi species in Co and Cu biosorption from synthetic aqueous solutions and the influencing factors (initial concentration of solution, biomass dose, pH, incubation time, temperature) were recently reviewed [25]. However, to the best of the author’s knowledge, no other review dealing exclusively with Co biosorption has been published to date.

In light of the above, the main goal of this work is to provide a useful tool for a step forward to cleaner removal, recovery, and determination of Co(II) in real-world conditions, by gathering together, for the first time, information on the separation of Co(II) from diluted aqueous solutions on biosorbents. Unlike the current scenario in the studies on heavy metals biosorption, this review is different because it discusses the biosorption features of polymeric biomasses on two levels, that is, as potential biosorbents and practical biosorbents for efficient separation/preconcentration of Co(II) with environmental, economic and analytical relevance. The main issues addressed are related to: (i) biosorbents for batch and fixed-bed column removal of Co(II) from mono-element synthetic solutions; (ii) biosorbents for Co(II) uptake from multi-component synthetic solutions; (iii) biological sorbents for analytical preconcentration of Co(II) from diluted synthetic solutions; (iv) real applications of biosorbents to Co(II) removal/recovery; (v) analytical procedures based on biosorbents for trace Co(II) determination from real samples.

## 2. Biosorbents Are Recommended as Promising Candidates for Co(II) Separation from Synthetic Solutions

### 2.1. Biosorption Capabilities of Polymeric Biomass

Biosorption is a green multidimensional process of metal retention from aqueous solutions on biological materials or materials derived from biological sources [32,48]. The irreplaceable advantages of the biosorption process include variety, variability, wide availability of eco-polymeric materials, its high efficiency for large volumes of wastewaters and very low concentrations, easy procedure, short operational time, versatility, low pollution, and low cost. The bio-based materials with biosorption ability can be categorized into three main classes: (i) dead biomass of microorganisms; (ii) agro-industrial wastes; (iii) other polysaccharide materials (chitin, chitosan, alginate) [37,49,50,51,52]. The first two categories of biosorbents have been most intensively investigated from the perspective of their integration in strategies for the bioseparation of heavy metals from real samples (Table 1). Contrasting with conventional adsorbents that contain a single kind of binding site, the biosorbents in Table 1 are rich in miscellaneous functional groups with multiple potentialities of binding, allowing for high retention of metals by a variety of mechanisms [35,36,37,38,39,40]. Moreover, the biosorbents stand out for their high bio-preconcentration potential, multifaceted applications, adaptability to batch and continuous (fixed-bed columns) systems, and their ability to work on the 3R principles (reduce, recycle, reuse) that govern the circular economy. 

However, the biosorption performances of the biomasses’ native forms in Table 1 do not, in many cases, fall within the coordinates of practical applicability. One solution to this problem was to apply a method of immobilization, modification, or immobilization/modification that folds on the desired improvement of biosorbent surface characteristics and/or functionalities [39,69,70,71,72].

After these opening remarks, the reports in the literature on the biologically based polymeric materials proposed over time for the biosorption of Co(II) from synthetic solutions are systematized and debated in the next sections.

### 2.2. Biosorbents for Batch and Fixed-Bed Column Removal of Co(II) from Mono-Element Synthetic Solutions

The preference of biomaterials for the metal ion under study is mainly due to their high content in surface functional groups with oxygen as the donor atom (hydroxyl, carboxyl carbonyl, etc.) which are able to manage the Co(II) binding by interaction mechanisms of electrostatic interactions, ion exchange, complexation, and chelation. On the basis of FTIR spectral multivariate statistical analyses, biosorbents with the largest amounts of-OH of alcohols and C–H, C–O–C, C–N and P–O of polysaccharides were assumed to have a propensity to uptake Co(II) superior to other biomaterials [73].

The best part of the investigations on Co(II) biosorption was limited to lab batch biosorption studies briefly described in Table 2. They mainly address fundamental research. Due to the practical limitations of the batch operation mode, the biosorption technology transfer from the lab to the industrial scale is inconceivable without continuous fixed-bed column studies. The principal aspects targeted in the few studies dealing with dynamic biosorption of Co(II) are also presented in Table 2. Both types of biosorption experiments should be accompanied by desorption studies focusing on the selection of the best desorbing agent and the determination of the minimum number of cycles of biosorbent reusability.

Among the microorganisms reported as potential biosorbents for batch biosorption of Co(II) from mono-metallic aqueous solutions are the following: six species of green, brown, and red seaweed [74]; *Padina sanctae crucis* brown marine alga [75]; 2-*Hypnea Valentiae* alga [76]; *Synechocystis pevalekii* cyanobacterial alga [6]; fungi (*Trichoderma, Penicillium*, *Aspergillus, Geotrichum, Monilia*, *Fusarium* species) [25]; Gram-negative bacteria, including *Shewanella* spp. [77] and *Serratia marcencens* [78]. However, the microbial biosorbents are clearly outclassed by the agro-industrial wastes, which are addressed in about 75% of fundamental research studies on biosorption of Co(II). These attempts are in line with the current trend of converting waste into valuable and useful resources for sustainable development and advanced strategies of waste management. The waste biomaterials with promising applicability in batch Co(II) biosorption from synthetic mono-component aqueous solutions include: banana and orange peels [79]; black carrot (*Daucus carrota* L.) residues [80]; almond green hull [81]; corn silk [82]; *Amaranthus hydridus* L. stalk wastes [83]; agricultural waste *Luffa cylindrica* [84]; powders of groundnut seed cake, sesame seed cake and coconut cake [85]; forestry wastes of pine sawdust [86] and eucalyptus bark [87]; bones of animal [88], cuttlefish [89] and *Lates niloticus* fish [90]; biomass derived from the pulp of *Saccharum bengalese* [91]; *Chrysanthenum indicum* flower biomass [92]; dead neem leaves [93]; clearing nut seed powder [94]; sludge of sewage treatment plants [95,96,97].

**Figure 1:**
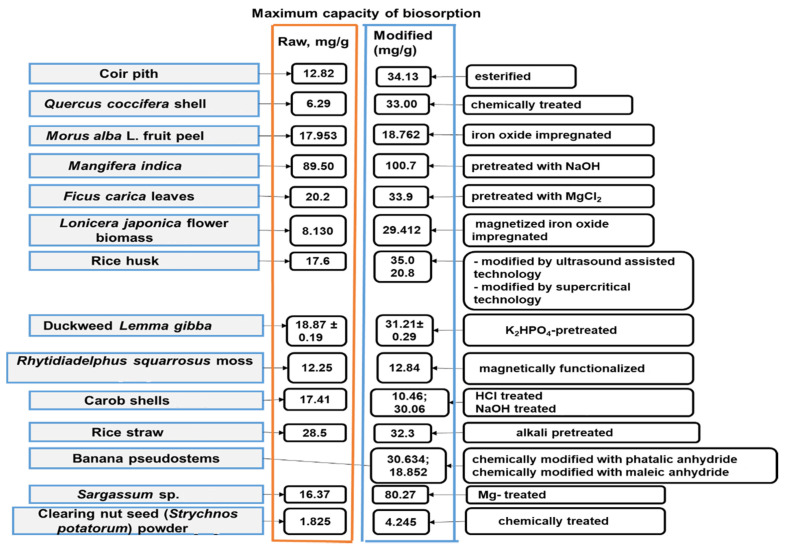
Comparison between raw and modified biomasses for batch removal of Co(II) from synthetic solutions [27,29,94,98,99,100,101,102,103,104,105,106,107,108,109,110].

Before their feasibility as agents of Co(II) decontamination was studied, the surface of many aforementioned biomass raw forms was chemically or magnetically modified. The most popular methods are the pretreatment of biomaterials via inorganic and organic chemical modifying agents, mainly applied for biomasses cleaning and the substantial increase of their biosorptive activity. To highlight this enhancement, Figure 1 juxtaposes the maximum capacity of Co(II) biosorption of the selected biosorbents based on untreated and modified biomasses. 

From Figure 1, it is obvious that the application of a chemical or magnetic modification gives an edge to modified biosorbents. Moreover, Figure 1 shows that the level of improvement in biomasses features for biosorption goals strongly depends on the nature of chemical modifications. Thus, ultrasound-assisted technology was reported as more effective than the supercritical CO_2_ technology for the increase in the rice husk biosorption potential [105]. Among the acid and alkaline pretreatments performed on the carob shell, the one with sodium hydroxide ensured a remarkable increase in the maximum capacity of Co(II) biosorption [108]. The same favorable effect of sodium hydroxide pretreatment compared to the other chemical modifications by means of hydrochloric acid, nitric acid, phosphoric acid, acetic acid, benzene, formaldehyde, and hydrogen peroxide was reported for *Mangifera indica* waste biomass [102].

The narrow group of biosorbents successfully used for the mono-component sorption of Co(II) in fixed-bed column systems encompasses: the brown algae *Sargassum wightii* [74] and *Sargassum glaucescens* [111]; green alga *Ulva reticulata* [112]; sunflower biomass [113]; *Chrysanthenum indicum* flower [114]; native *Tectona grandis* leaves [115] and spent leaves of green tea, peppermint tea, and chamomile [116]; *Ficus benghalenesis* L. [117]; chemically modified sugarcane bagasse by oxidation [118] and esterification [119]. The general finding is that the Co(II) concentrations studied in column mode were at a high mg/L range and no more than five biosorbents based on pretreated biomasses were tested. Thus, the removal efficiency of Co(II) in concentrations of 100 mg/L was reported as 40.7%, 78.65% and 79.4% in fixed-bed biosorption using *Ulva reticulata* [112], *Sargassum wightii* [74], and carboxylated sugarcane bagasse [119], respectively. The Thomas column capacity of Co(II) biosorption for spent tea leaves followed the trend: peppermint tea (59.7 mg/g) > green tea (25.2 mg/g) > chamomile (24.9 mg/g) [116]. The value of column biosorption capacity was 65.2% lower than in the batch systems based on oxidized sugarcane bagasse for Co(II) removal [118].

It is very well known that the deciding features for practical applicability of biosorbents with remediation purposes are biosorption capacity and recyclability. The literature scan revealed two opposite trends. On one hand, the uptake capacity is described in all reviewed papers in which biosorbents were proposed as eligible green polymeric materials for Co(II) removal from aqueous solutions. On the other hand, the evaluation of their regeneration and reuse by means of desorption studies is still seldom conducted. From this perspective, Table 3 displays the results of some studies on the batch and fixed-bed column biosorption of Co(II) chosen on the basis of the data available on both the uptake capacity and reusability of the investigated biomass. The description of these characteristics in Table 3 is conclusive of the promising suitability of the corresponding biosorbents in the treatment processes of real wastewaters laden with Co.

The research articles that covered the fundamental concepts of Co(II) biosorption given in Table 2 and the results of which were reported in Table 3 were carried out with synthetic laboratory solutions and process conditions that significantly varied from one experimental approach to another. Therefore, the comparisons between the biosorbents made in all parametric studies cannot be an accurate reference for the selection of the biosorbent that works in optimum conditions to perform the best efficiency of Co(II) removal. For such goals, the modeling and optimization of the process of Co(II) biosorption through the response surface methodology and artificial neural network methods may be very helpful [113,117,121]. In this context, the following findings for real applications should be considered:

- By applying the response surface methodology combined with the central composite design, the highest efficiency of Co(II) batch removal (~84.82%) from an aqueous solution of 10 mg Co/L was obtained with 15 g/L of *Cocos nucifera* leaf powder, in 70 min, at pH = 5 and 303 K [122];
- Following the same optimization method, the use of *Ficus benghalensis* leaf powder ensured the achievement of 98.73% removal of Co(II), under the following optimized batch conditions: initial concentration of Co(II) solution: 20 mg/L; biomass dose: 25 g/L; pH = 5; temperature: 303 K [123];
- Performing batch experiments based on the models of artificial neural networks and genetic programming, the biosorption of Co(II) on the Rafsanjan pistachio shell could be maximized up to 69.4%, at pH = 5, with an initial concentration of 10.2 mg/L of Co(II) solution, a biosorbent dose of 0.8 g/L and a temperature of 25° [124].

Future research prospects should target the following issues: (i) expand the range of biosorbents to be tested for Co(II) removal from aqueous solutions of concentrations that reflect industrial reality; (ii) investigate increasingly cleaner methods of biosorbent modification; (iii) conduct much more research on the fixed-bed column biosorption of Co(II); (iv) increase the number of desorption studies.

### 2.3. Biosorbents for Co(II) Uptake from Multi-Component Synthetic Solutions

The chemical composition complexity of real matrices brings into the foreground the relevance of the studies on multi-component biosorption of Co(II) for successful practical applications. The works on this topic, which are still very few, mainly consider the batch operation mode by addressing the following issues: (a) exploring the effects of heavy metal ions [76,78,98,125,126,127,128,129,130,131,132,133,134], light metal ions [92,108,135,136,137,138,139,140] and anions (nitrate, sulfate, carbonate, phosphate) [92,141,142] on the uptake of Co(II) by biosorbents; (b) evaluation of the biosorbents’ efficiency for the removal of Co(II) from synthetic complex multi-element solutions that simulate industrial effluents [26,73,77,98,137,143,144,145,146,147].

The significant reported results of the studies, which describe the impact of other metal ions on the behavior of biomaterials in the batch biosorption of Co(II) from multi-metal solutions, are recorded in Table 4. Most published papers refer only to batch binary systems in which Co(II) is associated with Cu(II), Zn(II), or Ni(II) ions, which are frequently present in industrial wastewaters. As can be seen from Table 4, the maximum capacity of Co(II) biosorption for each biosorbent in polymetallic solutions was lower than that obtained in the corresponding monometallic solution. This drop in biosorption capacity is caused by the competition between the metal ions for the active sites on the biosorbent. The competitiveness degree that dictates the level of uptake capacity decrease, as shown in Table 4, depends on the number and addition order of the metal ions, their physico-chemical features (atomic weight, charge, coordination number, electronic configuration, ionic radius, electronegativity), and their concentration [148,149,150]. However, the heterogeneity of the experimental conditions and methodological approaches, and data scarcity generally prevent valid conclusions from being drawn. Therefore, the results in Table 4 can be viewed as a basis for improving the current knowledge on competitive biosorption of Co(II) through further studies on increasingly complex solution.

Table 4 also shows the high degree of tolerance of biosorbents to multi-metal uptake. For this reason, the identification of biosorptive materials with relative selectivity for Co(II) that are able to ensure its removal under realistic conditions at a sufficiently low level is essential for biosorption development. Biosorbents suitable for such a purpose proved to be those based on fruit wastes. Thus, quantitative biosorptive removal of Co(II) from 50 mL of synthetic nuclear power plant coolant water sample, with 1 mg/L of Co(II), 4 mg/L of Cr(III), and 15 mg/L of Ni(II) in its composition, was reached at pH 4.6 with coir pith (100 mg) [98]. The successful treatment of 320 L of synthetic wastewater containing low concentrations of Co(II) and different other ions, by means of 1 kg of alkali-treated lemon peels, was reported [137]. Another biosorbent based on NaOH-treated lemon peel (300 mg) was removed, under batch conditions (pH = 5; 60 min; room temperature) 70.98% of the Co(II) from 50 mL of seven metal solution with a total concentration of 350 mg/L [145]. The percentage of batch biosorption of Co(II) from 100 mL of a synthetic solution containing 5 ppm of Cr, Cu, Mn, Co, Ni, Pb, and 2 ppm of Cd and Zn by chemically modified tangerine peel was 94.70% (pH = 5; 20 min; 300 mg of biosorbent) [146].

In order to extend the real applicability of the biosorptive separation of Co(II), emphasis should be placed, in particular, on a thorough understanding of the complicated interactions and dependences characteristic of multi-component systems of biosorption.

### 2.4. Biological Sorbents for Analytical Preconcentration of Co(II) from Diluted Synthetic Solutions

The quantification of Co(II) is of major importance for areas, such as environmental monitoring, quality, and process control, agriculture, medicine, etc. However, the direct determination of Co(II) by a given analytical method is, in many cases, very difficult or even impossible. This is due to its very low concentrations and the high content of interfering components of real matrices [151]. A preconcentration step prior to the measurement process is very useful for overcoming these limitations and improving the analytical performances of the determination methods.

In the current trend of analytical protocols greening, the preconcentration by solid-phase extraction based on the biosorption of trace heavy metals from various matrices was proposed as one of the best options [152]. According to this scheme, the research on the function of biosorbents as analytical preconcentrators for Co(II) quantification is of growing interest. Only one of the reported Co(II) bio-preconcentration procedures successfully incorporated sawdust pretreated with sodium hydroxide in the batch biosorption system, preceding its determination by flame atomic absorption spectrometry [153]. All the other studies were performed in continuous mode, focusing on the investigation of the quantitative biosorption conditions of Co(II) (effect of solution pH, type, flow rate, and volume of eluent, flow rate, and volume of sample, matrix influences, etc.) and the application of the proposed biosorption procedure to Co(II) determination from real samples. Moving forward, the studied biosorbents and their properties of analytical usefulness will be under consideration. The favorable effect of these features of biosorbents on the analytical merits and practical applicability of the developed methods will be underlined in the subsequent part of this review.

Pulverized banana peel [154] and pulverized peel of unmodified and modified pumpkin (*Cucurbita pepo* L.) [155] proved to be efficient biosorbents for the flame atomic absorption spectrometry determination of Co(II). Biosorbents based on pine sawdust and malt sprouts modified with orthophosphoric acid and carbamide were introduced for the preconcentration of Co(II) combined with determination by inductively coupled plasma optical emission spectrometry [156]. However, the solution of choice is represented by the microorganisms, especially bacteria and fungi, immobilized on solid supports [70]. Although *Pilayella littoralis* immobilized on silica gel [157] and *Penicillium digitatum* loaded on pumice stone [158] were proposed for the analytical preconcentrations of Co(II), the most targeted inert supports for biomass immobilization were synthetic resins. Among these, Amberlite XAD-4 resin was one of the most selected. The popularity of immobilized microorganisms is because a proper combination of a biological and supporting material provides the opportunity to tune the characteristics of the Co(II) biosorbents for analytical purposes. For instance, the values of analytical recovery of Co(II) at pH = 6 were reported to be 100%, <80%, and <40% on a column filled with *Bacillus sphaericus* loaded Diaion SP-850 resin, Diaion SP-850, and *Bacillus sphaericus*, respectively [159]. The sequence of analytical recovery percentage of Co(II) after fixed-bed column biosorption was in the following order: *Aspergillus fumigatus* immobilized Diaion HP-2MG > Diaion HP-2MG without *Aspergillus fumigatus > Aspergillus fumigatus* without Diaion HP-2MG [160]. In addition, the use of immobilized microorganisms, such as *Penicillium italicum* immobilized on Sepabeads SP 70 [161], *Bacillus thuringiensis var. israelensis* immobilized on Chromosorb 101 [162], and *Pleurotus eryngii* immobilized on Amberlite XAD-16 [163] in continuous flow procedures, eliminated the need to use chelating/complexing agents, Co(II) being preconcentrated directly.

Table 5 characterizes the fixed-bed column systems based on immobilized bacterial and fungal biomasses that reported the optimal value of the pH of sample solution for Co(II) analysis of about 8. To provide a reliable description, the selection in Table 5 was made by corroborating the importance of the pH of the solution on the biosorption process with the finding that most of the Co(II) biosorbents recommended for analytical utilization achieved optimum performances at the initial solution pH = 8. Besides the reasonable uptake capacity, the immobilized microorganisms in Table 5 are propitious by the low degree to which they are subject to interferences from possible matrix components and high stability for repeated use.

In recent years, there has been little work on fungal magnetized biomasses, such as *Boletus edulis* loaded with γ-Fe_2_O_3_ magnetized nanoparticles [169] and *Coprinus* *micaceus* immobilized on Fe_2_O_3_ magnetic nanoparticle [170], described as viable biosorbents for preconcentration of trace levels of Co(II), aiming its determination by inductively coupled plasma optical emission spectrometry. This appears to be a promising area for future study, along with further research on immobilized biomasses to improve their selectivity, the use of sustainable and biodegradable materials as support, and assess the analytical potential of more and more microorganisms.

## 3. Biosorbents Integrated into Practical Approaches for Removal/Recovery and Determination of Co(II) from Real Samples

The biosorption feasibility, as a cleaner alternative to conventional methods of separation–preconcentration, notably depends on the degree to which the biosorbents are able to access all specific requirements for realistic circumstances. For decontamination and recycling purposes, these are represented by high uptake capacity, selectivity, and efficiency, good stability, favorable kinetics, tolerance to a broad spectrum of environmental conditions, advanced regenerability and reusability, easiness in separation, and adaptableness to systems of different designs [24,33,36,40,52,171,172]. Besides a significant level of the biosorption capacity, selectivity, and stability, the biological sorbents for practical analytical goals should present good surface contact with the processed solution, high values of the distribution coefficient for the metal under study, quick quantitative biosorption–desorption, and tolerance to high flow rates in column procedures [35,41,46,52,152]. Despite the effervescence from the biosorption research field, the foregoing dependence, and the biosorption suitability of actual matrices are still very little known.

**Figure 2:**
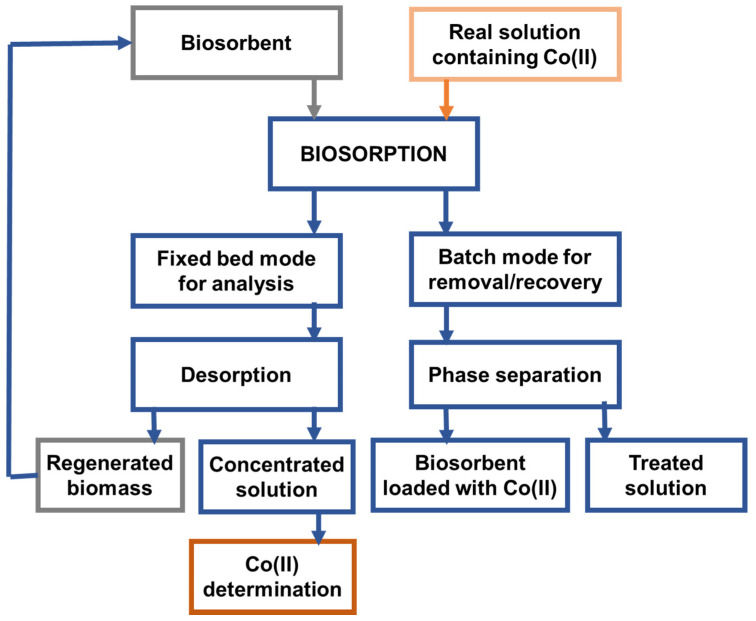
Schematic representation of the main biosorption-based procedures reported in the literature for removal, recovery, and analysis of Co(II) from actual matrices.

Similar to all other reports on the practical applications of the separation of heavy metal ions from real samples by using green biosorptive materials, those targeting Co(II) are in their pioneering phase. The aspects that have been tackled to date recorded practical approaches, schematically described in Figure 2, will be further discussed. As can be seen from Figure 2, the core of the developed strategies was the biosorption-based preconcentration of Co(II) from real solutions via batch procedures for metal remediation and in continuous fixed-bed column mode for Co(II) quantification. While the efficiency of the biosorption process aiming at Co(II) removal from real effluents has been under investigation, the recyclability of biosorbents in real industrial conditions has been scarcely studied. Instead, in analytical methodologies, the biosorption of Co(II) from large volumes of real matrices goes hand in hand with its desorption and determination in small volumes of concentrated desorption solution by an adequate method of instrumental analysis.

### 3.1. Real Applications of Biosorbents to Co(II) Removal/Recovery

Guided by the remediation or recovery purpose of the biosorption process’ practical applicability, two types of real matrices have been tested: wastewaters and leached solutions of lithium-ion batteries.

The confined information available for the biosorption removal of Co(II) from real wastewaters is depicted in Table 6. Because the investigations were done in batch mode, the significance of the studies in Table 6 is restricted to small amounts of wastewaters. Taking into account the high metal loading of real effluents in Table 6 and their Co concentrations ranging from 0.005 mg/L to 20 mg/L, the efficiency of the tested biosorbents in removing Co(II), along with other heavy metals, is distinguished. The performances of bisorbents are also reflected in the contact time values in Table 6.

There are very few considerations related to the compatibility of the biosorbents with the real systems of treatment of wastewaters containing Co from reusability and cost viewpoints. Hence, it has been demonstrated that the efficiency of the Co(II) biosorption from industrial wastewater on regenerated algal biomasses of *Corralina mediterranea, Galaxaura oblongata, Jania rubens,* and *Ptredocladia papillacea* was almost unchanged for two consecutive cycles [174]. A cost estimation indicated that peanut husk powder used for the treatment of a real effluent [175] was 5 times cheaper than another biosorbent based on lemon peel proposed for Co(II) removal from synthetic wastewater [181] and 50 times cheaper than the commercial activated carbon.

The only two batch studies with real leachates of lithium-ion batteries processed by biosorbents can be considered concept proof. They reported high percentages of Co(II) recovery by means of waste biomass [182] and dried algal biomass [183]. Under optimum batch conditions (pH = 6, 4 h contact time, 318 K), a dose of 10 g/L of chitin (seafood industry waste) was able to recover 95% of Co(II) from 50 mL of real leached solution with a Co concentration of 98.3 ± 5.1 mg/L and a Li concentration of 12.3 ± 3.6 mg/L [182]. Furthermore, 82% of the Co contained in real leachate (113.3 ± 4.9 mg/L) was recovered by *Spirulina* biosorption treatment, at an extremely acidic pH of 1 and in the presence of Li with a concentration of 20.2 ± 2.5 mg/L [183].

Apart from a drastic increase in the works on biosorptive removal and recovery of Co(II) from real matrices, the expected advances towards the practical applications strongly require pilot- and full-scale studies. Moreover, future research should be focused on addressing issues related to cost, energy requirements, desorption–regeneration with real effluents, and the disposal of exhausted biosorbents.

### 3.2. Analytical Procedures Based on Biosorbents for Trace Co(II) Determination from Real Samples

As previously demonstrated, the immobilized bacteria and fungi have the potential to be alternative tools of analytical preconcentration in fixed-bed column systems. The proposed procedures linked Co(II) enrichment by biomass, mostly with detection by inductively coupled plasma optical emission spectrometry or flame atomic absorption spectrometry. The reported values of the preconcentration factor ranged from 11 [157] to 111.1 [184]. This implies that the associated approaches have attractive analytical features since they satisfy the requirement that a method is only good if it achieves a preconcentration factor of at least 6 [185]. They were effectively applied to the Co(II) determination from environmental and food samples after being validated by the analysis of certified reference materials.

The transposition of biosorption potential in sustainable methodologies for Co(II) determination from actual samples of the above-named types is described in Table 7. To emphasize distinctive achievements, only the studies that conducted a comparative analysis of the developed procedures towards literature conventional preconcentration methods for Co(II) were systematized in Table 7. Against this background, the procedures in Table 7 were evaluated as having a lower limit of detection with higher preconcentration factors and a wider linear range. On the other hand, the relative standard deviation was, in many reports, in Table 7, less than 5%, being consistent with a satisfactory reproducibility of the process of Co(II) biosorption [42]. The systematized results in Table 7 also showed a very good correlation between the concentrations found for Co(II) and the certified values.

The prospects of research on this topic might be: (a) further refinement of the already proposed procedures; (b) the adjustment of more and more biosorption processes to the rigors of instrumental methods of analysis; (c) and a substantial broadening of the spectrum of real samples analyzed by means of biosorbents.

## 4. Conclusions

This review is focused on the ability of polymeric biomasses with evolved biosorption activity to carry out a triple task in the removal, recovery, and analysis of Co(II) from diluted aqueous solutions. According to the type of solution processed by means of Co(II) biosorbents, these were differentiated and reviewed as viable candidates for practical applicability and materials, ensuring good efficiency in real applications. Unfortunately, so far, the first group is much larger than the second. It primarily consists of biosorbents based on modified biomasses that performed very well in the removal of Co(II) from synthetic solutions, as well as immobilized bacteria and fungi with superior analytical features for Co(II) quantification. The results of the studies on the small number of the second group biosorbents provide evidence for the benefits of incorporating biosorption into practical strategies for the treatment and analysis of real waste solutions containing Co(II). In order to promote a significant change in the ratio between the member number of the two classes of biosorbents, researchers should concentrate their efforts on increasing continuous biosorption–desorption studies under competitive industrial conditions, expanding the range of processed real wastewaters and leached solutions, transitioning from laboratory tests to pilot-scale experiments, and performing economic analyses. More research on the valorization of the analytical potential of biosorbents for the development of eco-friendly methodologies of Co(II) determination from a wider range of actual samples is needed from an analytical standpoint.

## Figures and Tables

**Table 1:** Focus on the most targeted biosorbents of heavy metals.

Class of Biosorbents	Main Members	General Characteristics	Reference
Microorganisms Algae Fungi Bacteria	Marine macroalgae (seaweeds) - brown seaweeds; - red seaweeds; - green seaweeds - Micro-algae - diatoms; - green algae; - golden algae; - cyanobacteria	Cell walls are composed of chitin, polysaccharides, lipids, and proteins, in proportions dependent on the algae type; Excellent biosorption abilities for brown seaweeds, due to their alginate content in gel form; High surface to volume ratio; Large variety in shape and size; Capability of rapid biosorption.	[53,54,55,56]
	Molds Yeasts Mushrooms	Chemical composition of cell walls: polysaccharides (80–90%), heavily glycosylated proteins, lipids; Large proportion of material of cell wall over other biosorbents; Considerable resistance against low pH.	[57,58,59]
	Gram-positive Gram-negative	Functional groups involved in metal uptake: peptidoglycan, teichoic and teichuronic acids, phospholipids, lipopolysaccharides, proteins; Shape diversity and small size; Tolerance towards a wide range of environmental conditions.	[60,61,62]
Agro-industrial wastes Agricultural wastes Industrial wastes	- Husk, shells, steam, stalks; - Leaves; - Bran (rice, wheat); - Seeds, seed hulls, seed coat; - Fruit wastes; - Coir pith; - Fibers; - Sawdust of various plants, tree bark, etc.	Lignocellulosic materials consist of three main structural components: lignin, cellulose, and hemicelluloses; High surface area; Good porosity; Reasonable hardness; Low content of ash.	[63,64,65]
	- Waste biomass from food processing; - Pharmaceutical wastes; - Fermentation wastes; - Sugarcane bagasse; - Rapeseed cakes; - Sludge, sewage sludge	Specific physical features (surface area, porosity, stability) and chemical composition for each waste biomass; Minor processing before use as biosorbent; Potential leaching of some components.	[66,67,68]

**Table 2:** Outline of the batch and fixed-bed column studies regarding the removal of Co(II) from aqueous solutions by biosorbents.

	Targeted Issue	Summary of Common Findings
Batch studies (mixing of a small amount of biomass with a certain volume of Co(II) solution→ biosorption→ separation of used biomass)	Assessment of the biosorbent affinity for Co as a function of the most feasible parameters of the process: pH of the initial solution; dose of biosorbent; the initial concentration of metal solution; contact time; temperature.	Initial pH of solution plays the protagonist role in Co(II) uptake on the reviewed biosorbents. In most cases, Co(II) biosorption: - is reduced at low pH values; - increase with the increasing of initial pH; - reaches its maximum at pH values ranging from 5 to 6 depending on biosorbent nature.
	Biosorption interactions quantification and prediction of biosorption capacity by equilibrium modeling (models of Langmuir, Freundlich, Redlich–Peterson, Dubinin–Radushkevich, Temkin isotherms)	The reported processes of Co (II) biosorption followed Langmuir isotherm model, highlighting their monolayer character. Maximum capacity of biosorption provided by means of Langmuir isotherm is the basis of biosorbent performances appraisal.
	Uptake rate determination and biosorption mechanism understanding by kinetic modeling (pseudo-first-order model, pseudo-second-order, diffusion models)	The pseudo-second-order model has been the best-fit kinetic model, meaning that chemisorption is predominant in the mechanism of Co(II) biosorption.
	Predicting of biosorption process nature by means of thermodynamic parameters evaluation	Biosorptive removal of Co(II) has been frequently reported as being endothermic and spontaneous.
Fixed-bed column studies (Co(II) solution continuously flows through a biomass bed at a constant rate)	Analysis of fixed-bed biosorption variables by means of breakthrough curves	Most researchers have worked on the effect of flow rate, bed height, and metal solution initial concentration on the fixed-bed column biosorption of Co(II) from synthetic solutions.
	Modeling of breakthrough curves (Thomas, Yoon–Nelson, Bohart–Adams, bed depth service time models)	The large majority of experimental breakthrough data have been very well described by the Thomas model.

**Table 3:** Selected potential biosorbents for Co(II) sequestering from mono-element aqueous solutions.

Biosorbent; Reference	Biosorption Operation Mode; Working Conditions	Biosorption Capacity	Recyclability		
			Desorbing Agent	Desorption Efficiency (%)	Number of Cycles
Brown alga *Sargassum wightii*; [74]	Batch mode: pH = 4.5, 0.2 g of biomass, contact time: 12 h Fixed bed column: flow rate of 5 mL/min, bed height of 25 cm	20.63 mg/g 46.08–50.69 mg/g	0.1 M CaCl_2_ (in HCl)	99.39–98.42 98.4–99.2	5 5
Corn silk modified by diluted nitric acid; [82]	Batch mode: pH = 6; 20 mg of biomass, contact time: 20 min	90.09 mg/g	0.5 M HNO_3_	98.33 ± 0.4	at least 11
Bark of eucalyptus grafted with acrylic acid; [87]	Batch mode: pH = 6; 0.2 g of biosorbent, 100 mL of sample	55.55 mg/g	0.1 M HNO_3_	71.6–69.91	3
Chemically modified *Sargassum* *glaucescens*; [111]	Fixed bed column: flow rate of 7 mL/min, pH = 4, bed height: 30 cm	27.6 mg/g	0.1 M CaCl_2_; pH = 3		4
Green alga *Ulva reticulata*; [112]	Fixed bed column: flow rate: 5 mL/min; pH = 4; bed height: 25 cm	46.1 ± 0.07 mg/g	0.1 M CaCl_2_ at pH 3 adjusted with HCl	99.9–99.2	3
*Chrysanthenum* *indicum* flower; [114]	Fixed bed column: 1 mL/min flow rate, pH = 5, 1 cm bed height	14.84 mg/g	0.1 M HCl	76.1–66.7	4
*Tectona grandis* leaves; [115]	Fixed bed column: 1 mL/min flow rate, 1 cm bed height	23.48 mg/g	0.1 M HCl	79.8–65.5	4
Sugarcane bagasse - oxidized; [118] - carboxylated; [119]	Batch: biosorbent dose: 2 g/L, pH = 5.5, contact time: 4 h Fixed bed column: 5 mL/min flow rate; 1.679 mmol/L initial concentration	0.37 mmol/g 0.782 mmol/g	0.5 M HNO_3_ 0.01 M HNO_3_	98.1–85.3 95	2 3
K_2_HPO_4_-pretreated duckweed *Lemma gibba*; [120]	Batch: pH = 7, biosorbent dose: 1 g/L, contact time: 30 min	46.17 ± 0.41 mg/g	0.1 M HCl	100	3

**Table 4:** Batch biosorption systems for Co(II) retention from polymetallic synthetic solutions.

Biosorbent; Reference	Composition of Multi-Metal Solution	Working Conditions			Maximum Capacity of Co(II) Biosorption (mg/g) in		Comments
		pH	Biomass Dose (g/L)	Contact Time (Min)	Tested Multi-metal Solution	Single- Metal Solution	
Formaldehyde treated 2-*Hypnea Valentiae* alga; [76]	Co(II) + Ni(II) Co(II) + Zn(II)	6	2	120	~23.72 ~46.49	47.44	Internal competition with H_3_O^+^ and the other ions for surface active sites
Cyanobacteria *Oscillatoria* *Angustissima*; [127]	Co(II) + Cu(II) Co(II) + Zn(II) Co(II) + Cu(II) + Zn(II)	4	1	60	15.91 14.14 5.30	24.75	Trend of affinity series: Cu > Co >Zn
Aerobic granules; [128]	Co(II) + Zn(II)	7	0.1	150	54.05	55.25	Order of initial biosorption rate: Co > Zn
Watermelon rind; [129]	Co(II) + Ni(II) Co(II) + Cu(II) Co(II) + Cd(II) Co(II) + Zn(II) Co(II) + Ni(II) + Cu(II) + Cd(II) + Zn(II)	5	2	30	6.8 6.5 5.7 9.9 1.3	10.2	Decrease of biosorption capacity by 35–40% Drop of biosorption capacity up to 90%
Pretreated *Saccharomyces* *cerevisiae* immobilized with polysulfone polymer; [130]	Co(II) + Ni(II) + Cd(II)	8	8	80	0.61	1.768	Sequence of metal biosorption: Co > Ni > Cd
Sugarcane bagasse - carboxylated; [131] - phatalate functionalized; [132]	Co(II) + Cu(II) Co(II) + Ni(II) Co(II) + Cu(II) Co(II) + Ni(II)	5.5	0.2	180–250	14.496 21.686 8.957 10.607	67.180 33.059	Order of maximum biosorption capacities: Cu > Ni > Co
Arborvitae leaves; [133]	Co(II) + Pb(II) + Cu(II)	5.5	0.1	300	1.54	6.78	Biosorption affinity order: Pb > Cu > Co
Sulfate reducing bacteria biomass; [135]	Cs(I) + Co(II) Sr(II) + Co(II)	4	0.5		49.3 185.2	204.1	Possible existence of specialized sites for Co binding
Biomass of moss *Rhytidiadelphus squarrosus*; [136]	Co(II) + Sr(II)	6	2.5	240	5.84	7.25	Larger affinity against Co(II) compared to Sr(II)
Lemon peels -raw and -alkali treated; [137]	Co(II) + Ca(II) Co(II) + Mg(II) Co(II) + Ca(II) Co(II) + Mg(II)	6	2	150 – 210	19.18 17.86 32.89 30.64	20.83 35.71	Significant effect on the Co(II) biosorption capacity at 100 mg/L addition of cations
Macroalgae: *Ulpia fasciata* *Colpomenia* *sinuosa*; [138]	Co(II) + Ca(II) Co(II) + Na(I) Co(II) + Mg(II) Co(II) + Na(I)	6 7	10	60	1.24 1.91 0.97 2.82	3.12 3.08	Foreign ions effect: Ca > Mg > Na Mg > Ca > Na

**Table 5:** Biosorbents based on immobilized microorganisms for analytical column preconcentration of Co(II) from model solutions of pH = 8.

Biosorbent				Capacity of Co(II) Biosorption	Foreign Ions without Major Interference effects on Co(II) Retention and the Reported Tolerance Limits	Number of Reused Cycles	Reference
Microorganism	Support for Biomass Immobilization	Optimum Amount of					
		Biomass	Support				
*Aspergillus* *fumigatus* *Anoxybacillus gonensis*	Diaion HP-2MG	150 mg 125 mg	1 g 1 g	4.4 mg/g 6.16 ± 0.2 mg/g	Na^+^ (20 g/L); K^+^ (5 g/L); Ca^2+^, Mg^2+^, F^–^, NO_3_^–^, SO_4_^2–^ (2 g/L); Al^3+^, Cr^3+^ (10 mg/L); Mn^2+^, Cd^2+^ (25 mg/L) Na^+^ (10 g/L); Ca^2+^, Mg^2+^, SO_4_^2–^, NO_3_^–^ (1 g/L); Al^3+^, Mo^6+^, Cr^3+^, Hg^2+^ (10 mg/L)	>50 50	[160] [164]
*Escherichia coli* *Saccharomyces carlsbergensis* *Agrobacterium* *tumefacients*	Amberlite XAD-4	150 mg 200 mg 150 mg	1 g 1 g 1 g	28 µmol/g 24 µmol/g 29 µmol/g	Na^+^, K^+^ up to 500 µg/mL Na^+^, K^+^ up to 500 µg/mL Na^+^, K^+^, Al^3+^ up to 500 µg/mL	Up to 15 15 10	[165,166,167]
*Escherichia coli*	Multiwalled carbon nanotubes	0.1 g	0.1 g	0.072 mmol/g	Na^+^ (1150 µg/mL); Mg^2+^ (253 µg/mL); K^+^ (523 µg/mL); NH_4_^+^ (336 µg/mL); SO_4_^2–^ (676 µg/mL)	50	[168]

**Table 6:** Summary of the reports on the treatment of real wastewaters containing Co(II) by using biosorbents.

Type of Real Effluent; Reference	Co(II) Concentration (mg/L)	Other Elements Contained in Waste Solution (mg/L)	Biosorbent	Operating Conditions	Efficiency of the Process of Co(II) Biosorption	Remarks
2 samples of industrial wastewater; [109]	0.0543 0.112	Fe (2.954) Cu (1.564) Ni (0.1524) Cd (0.1201) Pb (0.0974) Fe (3.157) Cu (1.346) Ni (0.112) Cd (0.1674) Pb (0.1043)	Rice straw Modified rice straw	pH = 6.3; biomass dose: 0.4 g/50 mL; contact time: 1.5 h; temperature: 30 °C, 40 °C, 50 °C	100% 100%	Efficiency of other metals removal: 100% Complete removal of other heavy metals
Steel and electroplating industry effluents; [173]	0.58	Cr(III) (20.22) Cu (9.24) Fe(III) (1.08) Cd (0.73) Pb (2.06) Zn (5.8) Ag (1.02)	Dead biomass of *Geobacillus* *thermodenitrificans*	pH = 6.5; 25 mL of sample; 120 min contact time; 50 mg of biomass	Up to 11.43% reduction of Co(II) concentration	Order of biosorbent preference: Fe > Cr > Cd > Pb > Cu > Co > Zn > Ag
Effluent from chemical production; [174]	1.34	Cd (1.21) Cr (0.72) Pb (0.68)	*Corralina* *mediterranea* *Galaxaura* *oblongata* *Jania rubens* *Ptredocladia* *papillacea*	pH = 5; 60 min contact time; biomass dose: 10 g/L	86.2% 87.6% 90.6% 95.3%	Mean biosorption efficiency 84%
Industrial wastewater collected from a metal industry; [175]	20	Pb (0.26) Zn (11.61) Cu (11.55) Fe(III) (2.13) Ni (30.76) Cd (46) Mn (52) Cr (44.60)	Peanut husk powder	pH ~ 6.6 biosorbent dose: 5 g/L 1 h contact time	30%	Removal efficiency of other metals ranging from 24% for Ni to 100% for Pb
Wastewater samples from sewage treatment plant; [176]	0.342 ± 0.0023	Ni (0.271)	Vinegar-treated eggshell waste biomass	pH = 7.49; 77.41 mg of biomass; 50 mL of sample; 64.81 min contact time	76.53 ± 1.21%	78.7 ± 1.02 percentage of Ni(II) removal
Acidic and alkaline effluents from battery industry; [177]	0.16 0.05	Ni (0.43) Zn (0.82) Cd (84.32) Fe (1.83) Pb (2.05) Sb (0.23) Cu (0.1) Ni (1.132) Zn (17.78) Cd (0.02) Pb (5.37) Sb (0.16) Cu (0.03)	Dried activated tannery sludge	pH = 5.3; 0.2 g of biomass; 24 h contact time	75% 80%	% biosorption of other metals: 8.69 (Sb)- 96.74 (Ni) % biosorption of other metals: 33.33 (Cu)- 97.3 (Zn)
Wastewater collected from plating plant; [178]	8 ± 3	Ni (19 ± 4) Cr(VI) (14.5 ± 3) Zn (12 ± 3)	*Aspergillus flavus* modified by calcium chloride	pH = 5.5; 150 mL of sample; biomass dose: 4 g/L; contact time: 60 min	Non-detectable concentration of Co(II) after treatment	Significant decrease of Ni and Cr content after biosorption; Zn–non-detectable
Industrial wastewater; [179]	0.005 0.015	Pb (0.01) Cu (0.02)	Calcified *Solamnen* *Vailanti* snail shell	pH = 6; biomass dose: 2 g/L; contact time: 60 min; temperature: 25 °C	74% 84%	Removal efficiency of 85% and 91% for Pb and Cu, respectively
Industrial effluent; [180]	1.621	Ni (1.17) Cu (0.663) Zn (1.988) Cr (0.55) Al (1.611) Fe (1.666) Sn (0.23) Cd (<0.002) Mn (10.1) Ti (0.026)	Hemp felt Modified hemp felt	pH = 7.5; 15 g of felt; 15 L of wastewater; contact time: 30 min; 20 ± 1 °C temperature	Co concentration after treatment: 0.36 mg/L 0.003 mg/L	Ability of modified hemp felt to remove 80–100% of the total metal load

**Table 7:** Studies on the determination of Co(II) from real samples based on column biosorptive preconcentration in conjunction with instrumental analysis.

Processed Sample; Reference	Biosorbent; Maximum Capacity of Biosorption	Working Conditions			Desorption Agent; Detection	Analytical Performances of the Proposed Method
		Flow Rate (mL/ min)	Applicable Volume of Sample Solution (mL)	pH		
Spiked water and food samples and 2 certified reference materials; [169]	*Boletus edulis* immobilized γ-Fe_2_O_3_ magnetized nanoparticles; 35.8 mg/g	3	50–500	6	1 M HCl; inductively coupled plasma optical emission spectrometry	Detection limit: 0.021 ng/mL Preconcentration factor: 100 Linear range: 0.2–10 ng/mL Relative standard deviation: 4.9%
Water and food samples and 4 certified reference materials; [170]	*Coprinus micaceus* loaded with γ-Fe_2_O_3_ magnetized nanoparticles; 24.7 mg/g	3	Up to 400	5	1 M HCl; inductively coupled plasma optical emission spectrometry	Detection limit: 0.017 ng/mL Preconcentration factor: 80 Linear range: 0.25–12.5 ng/mL
Sample of Ontario lake water and reference standard material; [184]	Ostracod carapace of *Herpetocypris brevicaudata* loaded on Amberlite XAD-4 resin; 13.55 mg/g	5	Up to 1000	10 ± 0.1	1 M HCl; UV-VIS spectrophotometry	Detection limit: 1.4 µg/L Relative standard deviation: <5% Preconcentration factor: 111.1
Boiled wheat, canned fish, black tea, and lichen and sample of certified reference materials; [186]	*Pseudomonas* *aeruginosa* immobilized on multiwalled carbon nanotubes; 6.06 mg/g	5	25–500	9	1 M HNO_3_; flame atomic absorption spectrometry	Detection limit: 0.74 µg/L Preconcentration factor: 50
Natural water samples and 4 certified reference materials; [187]	*Pleurotus eryngii* loaded Fe_2_O_3_ magnetic nanoparticles; 25.4 mg/g	2	400	5	1 M HCl; inductively coupled plasma optical emission spectrometry	Detection limit: 0.014 ng/mL Linear range: 0.25–12.5 ng/mL Preconcentration factor: 80
Tap, sea, and dam water samples and sample of a certified reference material; [188]	Resting eggs of aquatic creatures living in freshwater; 46.0 ± 2.7 mg/g	4	25–2000	9	1 M HNO_3_; flame atomic absorption spectrometry	Detection limit: 41.4 µg/L Preconcentration factor: 67 Relative standard deviation: <4.1%
Water and food samples and certified reference material sample; [189]	*Bacillus altitudinis* immoblilized on nanodiamond; 26.4 mg/g	3	25–400	5	1 M HCl; inductively coupled plasma optical emission spectrometry	Detection limit: 0.023 ng/mL Preconcentration factor: 80 Linear range: 0.25–12.5 ng/mL Relative standard deviation: 4.4%
Food and environmental samples and 2 certified reference materials; [190]	*Geobacillus* *stearothermophilus* SO-20 loaded with Amberlite XAD-4; 21.6 mg/g	3	25–400	6	1 M HCl; inductively coupled plasma optical emission spectrometry	Detection limit: 0.022 ng/mL Preconcentration factor: 80 Linear range: 0.25–12.5 ng/mL
Tap, river, and mineral water samples, food samples; samples of 3 certified reference materials; [191]	*Anoxybacillus* *kestanboliensis* loaded Amberlite XAD-4 resin; 24.3 mg/g	2	400	5	1 M HCl; inductively coupled plasma optical emission spectrometry	Detection limit: 0.04 ng/mL Preconcentration factor: 80 Linear range: 0.25–12.5 ng/mL Relative standard deviation: <6.8%
Food and water samples and 4 certified reference materials; [192]	*Tricholoma* *populinum* loaded on Amberlite XAD-4 resin; 30.3 mg/kg	3	25–500	5	1 M HCl; inductively coupled plasma optical emission spectrometry	Detection limit: 0.2–15 ng/mL Preconcentration factor: 100 Relative standard deviation: <3%

## Data Availability

Not applicable.
